# Pneumothorax, Pneumomediastinum, and Cervical and Facial Massive Emphysema Secondary to Colonoscopy: A Rare Complication of Colonoscopy

**DOI:** 10.1155/2024/1140099

**Published:** 2024-06-25

**Authors:** Ruben Daniel Perez Lopez, Julian Vargas Flores, Lenin de Jesus Orbe Garibay, Hugo Fernando Narvaez Gonzalez

**Affiliations:** ^1^Universidad Nacional Autónoma de México, Mexico City, Mexico; ^2^Institute for Social Security and Services for State Workers, Mexico City, Mexico

## Abstract

**Background:**

Colonoscopy is a resource used for the diagnosis, treatment, and monitoring of benign and malignant colorectal pathologies. The incidence of perforation is 0.03%–0.65% in diagnostic colonoscopy; however, the incidence can be up to 10 times higher in therapeutic interventions, such as polypectomies, increasing the risk of complications up to 0.07–2.1%.

**Materials and methods:**

Case report of a 71-year-old female who presents a rare complication due to a perforation in the sigmoid which developed pneumoperitoneum, pneumomediastinum, pneumothorax, and massive subcutaneous emphysema as a complication of a diagnostic colonoscopy where a biopsy of a friable lesion was performed.

**Results:**

A 71-year-old female that went to the emergency room due to acute generalized abdominal colic spasm pain with a duration of 7 hours, associated with significant abdominal distension, malaise, diaphoresis, progressive dyspnea, and massive subcutaneous emphysema that developed after performing panendoscopy and colonoscopy for diverticulosis follow-up. An abdominal CT scan with double contrast was performed, reporting suggestive data of hollow viscus perforation, pneumoperitoneum, pneumomediastinum, pneumothorax, and massive subcutaneous emphysema in the thorax, neck, and skull base. She underwent an exploratory laparotomy finding a perforation in the sigmoid for which sigmoidectomy was performed, and for the pneumothorax and pneumomediastinum, endopleural tubes were placed in both hemithoraxes. The massive subcutaneous emphysema subsided with observation and oxygen.

**Conclusion:**

A rare complication of the use of colonoscopy as a diagnostic and therapeutic method is presented. The purpose of presenting this case is for the doctor who performs these interventions to suspect this complication in a timely manner, not delaying the diagnosis and carrying out an urgent therapeutic approach as in this case with exploratory laparotomy, finding the perforation site and carrying out the corresponding surgical management. We demonstrated that massive subcutaneous emphysema can be managed with observation if there is no other alarm data evident that required another surgical approach.

## 1. Introduction

Colonoscopy is a resource used for the diagnosis, treatment, and monitoring of benign and malignant colorectal pathologies [1]. While generally safe, like any medical intervention, it carries certain risks. Over the years, its use has increased; it is considered a procedure with a low risk of complications; however, when these occur, it can present hemorrhage, perforation, and cardiorespiratory compromise [1–4].

Three methods have been identified as potential causes of colonic perforation during colonoscopy: barotrauma, associated mechanical trauma, and associated therapeutic trauma [5].

The incidence of perforation is 0.03%–0.65% in diagnostic colonoscopy; however, the incidence can be up to 10 times higher in therapeutic interventions, such as polypectomies, increasing the risk of complications up to 0.07%–2.1% [2, 6].

Risk factors for colonic perforation include advanced age, female sex, comorbidities, low body mass index, low plasma albumin levels, inflammatory bowel disease, and acute diverticulitis, as well as therapeutic colonoscopy involving procedures such as polypectomy, dilation, endoscopic mucosal resection, and treatment of patients from the intensive care unit (ICU) [1, 7, 8].

We present the case of a patient with colon perforation with acute diverticulitis and subcutaneous emphysema with pneumoperitoneum, pneumomediastinum, and pneumothorax.

The objective of this work is to present a rare case of a complication due to colonoscopy with polypectomy which developed pneumoperitoneum, pneumothorax, pneumomediastinum, and cervical and facial emphysema and to make an association with the reported literature and its treatment options.

## 2. Materials and Methods

We present the case of a 71-year-old female with a history of systemic arterial hypertension diagnosed 3 years ago, diverticulosis diagnosed 4 years ago, laparoscopic cholecystectomy 3 years ago, and cesarean section 35 years ago secondary to lack of labor progression, allergic to paracetamol and amikacin.

**Figure 1:**
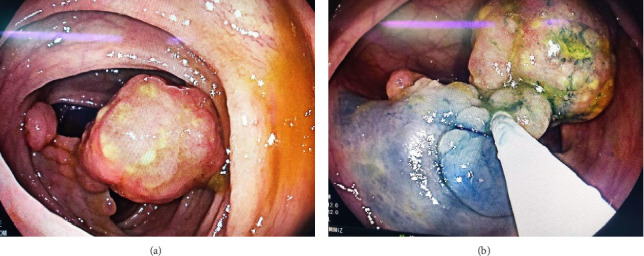
(a) Colonoscopy revealing a single pedunculated polyp, 10 mm in size, with an irregular and multilobulated shape, and heterogeneous color. (b) A hot snare polypectomy was performed without any incidents or complications.

She went to the emergency room due to acute generalized abdominal colic spasm pain with a duration of 7 hours, associated with significant abdominal distension, malaise, diaphoresis, progressive dyspnea, and massive subcutaneous emphysema that developed after performing panendoscopy and colonoscopy for diverticulosis follow-up. During the colonoscopy, a single pedunculated polyp was reported, measuring 10 mm in size, irregular and multilobulated in shape, and heterogeneous in color. A hot snare polypectomy was performed (Figures 1(a) and 1(b)).

**Figure 2:**
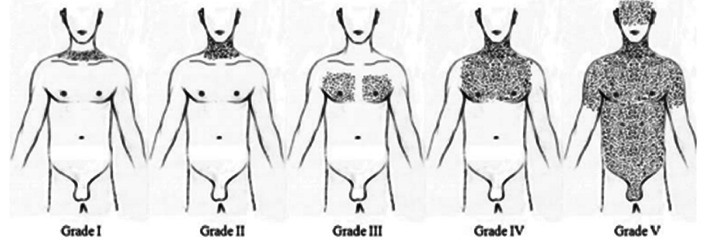
Classification of the severity of subcutaneous emphysema.

**Figure 3:**
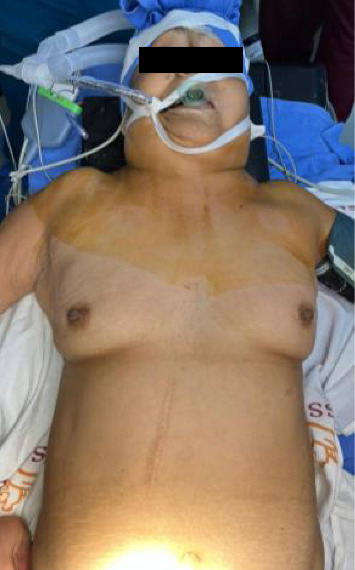
Subcutaneous emphysema in the thoracic, cervical, and facial regions.

Vital signs were taken, reporting hemodynamically unstable with the use of dose-response norepinephrine. On physical examination, subcutaneous emphysema was found from the facial region to the thoracic region Grade V of Aghajanzadeh [9] (Figure 2), with abdominal distention, aperistalsis, and nonspecific generalized abdominal pain, with signs of peritoneal irritation (Figure 3). Upon admission, laboratory tests were performed, finding leukocytes (Leuc) of 35.2 10 × 3/*μ*L, neutrophils (Neut) of 96.2%, hemoglobin (Hgb) 12 g/dL, hematocrit 37%, and platelets (Plt) 244,000 10 × 3/*μ*L.

**Figure 4:**
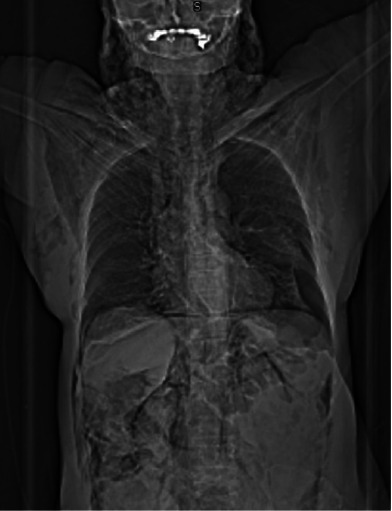
Topogram showing pneumoperitoneum, pneumoretroperitoneum, pneumomediastinum, pneumothorax, and massive subcutaneous emphysema.

**Figure 5:**
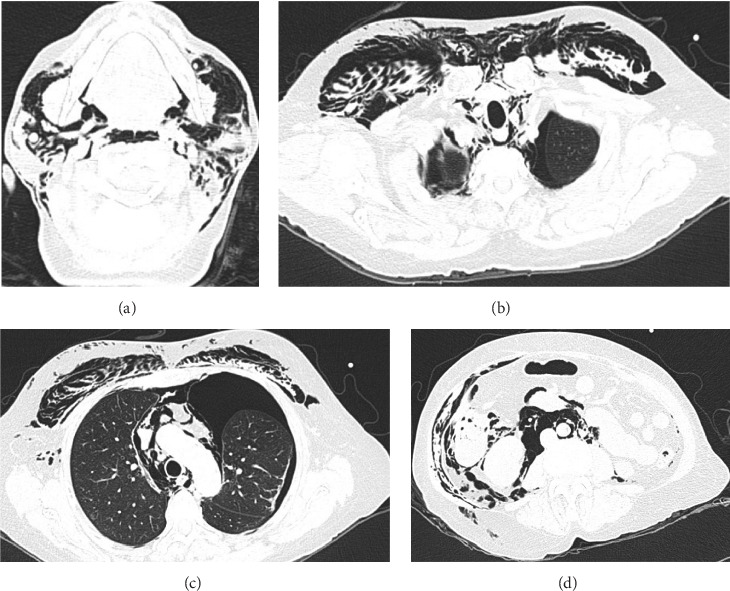
(a) Neck with multiple hypodense gas density areas located in soft tissues and muscular planes, from the base of the skull to the thorax. (b) Soft tissues with multiple gas density areas located towards the anterior thorax and in axillary regions and descending, gas density areas in the right renal fossa, as well as in the bilateral inguinal canal. (c) Mediastinum with the presence of multiple gas density areas of diffuse distribution is observed, with widening of the mediastinum, in the pleural space, in the right and left hemithorax with occupation by gas density images. (d) Sigmoid and rectum wall is observed with an increase in its diameter, with pneumatosis intestinalis, and with loss of interface in the wall of the distal segment of the sigmoid. Multiple areas of gas density, which are distributed throughout the retroperitoneum and peritoneum, and ascend to the diaphragmatic hiatus.

An abdominal CT scan with double contrast (computed axial tomography) was performed, where an increase in diameter was found in the distal sigmoid colon with intestinal pneumatosis, with a loss of interface and collapse of the same suggestive of hollow viscus perforation, pneumoperitoneum, pneumomediastinum, pneumothorax, and massive subcutaneous emphysema in the thorax, neck, and skull base (Figures 4, 5(a), 5(b), 5(c), and 5(d)).

Due to the CT findings, she was admitted to the operating room urgently, where she was evaluated by the cardiovascular surgery service, which placed 32 French (FR) endopleural tubes in both hemithoraxes in the 5th intercostal space in the anterior axillary line for decompression of pneumothorax.

**Figure 6:**
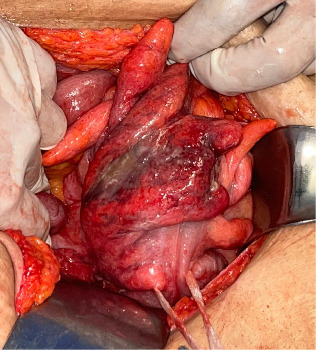
Perforation of 2 cm of diameter in the sigmoid in its distal segment on the right posterolateral wall.

General surgery performed an exploratory laparotomy, finding a perforation in the sigmoid in its distal segment on the right posterolateral wall of 2 cm of diameter, pneumatosis intestinalis, and emphysema infiltrating the mesosigmoid (Figure 6), for which sigmoidectomy was performed with closure in Hartmann's pouch and colostomy of the descending colon.

**Figure 7:**
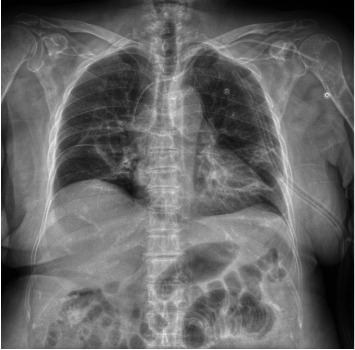
Simple chest X-ray showing adequate resolution of pneumothorax, pneumomediastinum, and decrease of the subcutaneous emphysema.

During evolution after surgery, norepinephrine was withdrawn due to the stabilization of vital signs on the third day after surgery, and oral enteral feeding was started on the fourth day after surgery with adequate tolerance. On the fourth day, a simple chest X-ray with a posteroanterior projection control was performed, finding adequate resolution of the pneumothorax and pneumomediastinum, even visualizing radiolucent areas in soft tissues suggestive of subcutaneous emphysema, and removal of the endopleural tubes was performed, without any eventualities (Figure 7). On the sixth postsurgical day, a significant decrease in massive emphysema in soft tissues was observed, as well as its resolution on the seventh day, which was managed with close monitoring. On the seventh day after surgery, the decision to discharge her from the hospital was made, with follow-up by the outpatient clinic.

## 3. Discussion

Perforation of the colon as a result of a colonoscopy is a rare but potentially serious complication of this medical procedure, with a risk reported in the literature of 0.2% in diagnostic colonoscopies and can be up to 10 times higher when therapeutic interventions are performed as polypectomies [1]. In this case report, a therapeutic colonoscopy involving a hot snare polypectomy was performed, which led to a perforation in the sigmoid colon. Despite the low morbidity rate, there may be serious life-threatening complications such as pulmonary embolism, congestive heart failure, sepsis, and death [5]. Most reports of this type of complication are secondary to barotrauma, thermal injury, and instrumental puncture of the intestinal wall by the tip of the endoscope or through interventions such as polypectomy and stricture dilation [1]. In this case, the perforation occurred in the sigmoid colon, in the context of documented diverticular disease. The Sigma is the anatomical portion of the colon most susceptible to perforation during endoscopy, since it can be exposed to excessive cutting forces of the endoscope, and it is also a common site for pathologies such as polyposis, massive lesions, and diverticulosis, which increase the probability of mechanical or thermal injuries in this particular area. The cecum, with its thinner wall, is the second most common site of perforation [1, 10].

Intraperitoneal perforations are the most common type. Ectopic gas can pass to different body compartments through different anatomical and fascial planes. The development of pneumomediastinum can occur for multiple reasons and is usually the result of an injury or an underlying disease. The thorax and abdomen originate from a single coelomic cavity during embryonic life. This cavity is lined by a serous membrane, and the deep space of this membrane is the subserous space. The coelomic cavity is divided into the peritoneal, pleural, and pericardial regions. In the adult, the serous membrane of the peritoneum is the peritoneal membrane, and the space beneath it is the subperitoneal or retroperitoneal space. The serous membrane of the chest becomes the pleura, and the deep space for this is the subpleural or mediastinal space. The continuity of the subserous space is maintained during the subdivision of the coelomic cavity, forming the thoracoabdominal duct that interconnects the subperitoneal (retroperitoneal) and subpleural (mediastinal) spaces [9, 11]. The esophageal diaphragmatic hiatus is located in the muscular part of the diaphragm at the vertebral level of T10 and admits the esophagus along with the anterior and posterior vagal trunks. It is connected to the bare area of the liver, which is also in communication with the gastrohepatic ligament and the retroperitoneum. This serves as a potential pathway for the spread of gas and various pathological processes between the retroperitoneum and the mediastinum. The diaphragmatic hiatus of the descending aorta is located at vertebral level T12, which admits the aorta into the retroperitoneum. There is, therefore, communication between the retroperitoneum and the mediastinum through the periaortic and paraesophageal fascial planes [1].

Therefore, the gas-generating pathological processes that follow these hiatuses and diaphragmatic defects will pass between the subperitoneal (retroperitoneal) and subpleural (mediastinum) spaces, providing a route between the retroperitoneum and the mediastinum. Once in the mediastinum, the rupture of the mediastinal pleura causes decompression of the gas in the pleural space, presenting as a pneumothorax [12].

The development of subcutaneous emphysema at the level of the chest and neck develops because there is no anatomical division or barrier between the subcutaneous cellular tissue in the body, and the subcutaneous cellular tissue acts as a path with no resistance for gas migration [1].

After the sigmoid perforation, gas may have traveled along the mesentery to the abdominal wall and then spread to the chest and abdominal walls and subcutaneous tissues of the neck.

In this case presented, the mechanism which we believe that the pneumoperitoneum was formed is due to the anatomical location of the intraperitoneal sigmoid since it is completely covered by the peritoneum.

Symptoms of perforation may appear after several hours. A review by Tiwari et al. revealed that 52% of perforations were detected immediately or within 1 hour, while 29% were found between 1 and 24 hours and 19% were found after 24 hours of the procedure [13, 14]. In the case presented, the symptoms occurred immediately after the colonoscopy with 7 hours of evolution before presenting to the emergency room. This means that the perforation in the sigmoid occurred during the hot snare polypectomy procedure and led to other complications in dissecting muscle planes.

In our clinical case presented, the diagnosis was made through the clinical history, revealing a history of colonoscopy with biopsy, time of development of symptoms, and the clinical presentation in the physical examination with crepitation on palpation from the abdomen, thorax, neck, and face in the bilateral malar region. An imaging test was performed with simple CT in which hypodense areas of diffuse distribution were evident in subcutaneous cellular tissue suggestive of massive subcutaneous emphysema, as well as pneumoperitoneum, pneumomediastinum, and bilateral pneumothorax. However, it was not possible to visualize the perforation site hollow viscera.

The treatment of this pathology is surgical to identify the perforation site and repair it. Management of subcutaneous emphysema should begin by identifying the cause of the subcutaneous air dissection. Several approaches have been described, including the use of subcutaneous infraclavicular incisions, needles, drains, or cervical mediastinotomy to accelerate the release of air from subcutaneous emphysema when there is evidence of compromise. Once massive subcutaneous emphysema is identified, it is classified according to severity with the classification proposed by Aghajanzadeh M et al. based on anatomical extent in five degrees, including (1) the base of the neck, (2) the entire neck area, (3) the subpectoralis major area, (4) the chest wall and the entire neck area, and the (5) chest wall, neck, orbit, scalp, abdominal wall, upper limbs, and scrotum (Figure 1).

Since treatment usually involves addressing the underlying condition, treatment is controversial; however, cases of subcutaneous emphysema may require nothing more than bed rest, pain control, and perhaps supplemental oxygen. Breathing oxygen can help the body absorb subcutaneous air more quickly. However, if subcutaneous emphysema generates dyspnea, respiratory distress, skin necrosis, alteration in visual acuity, or blindness secondary to eyelid edema, a surgical approach may be considered [15]. Just as in the case presented in which the surgical approach was performed exploratory laparotomy with sigmoidectomy to find and remove the perforation site because the intestinal wall had an important compromised surface. The pneumothorax was treated with the placement of endopleural tubes that improved in a short time and the massive subcutaneous emphysema subsided with observation and oxygen.

## 4. Conclusion

In this case report, a rare complication of the use of colonoscopy as a diagnostic and therapeutic method with hot snare polypectomy is presented, presenting as pneumoperitoneum, pneumothorax, and facial emphysema, highlighting the pathogenesis of the anatomical relationship by which emphysema migrates to different topographic levels. The case demonstrated risk factors for the complication such as female sex, advanced age, and hypoalbuminemia, according to the literature. The purpose of presenting this case is for the doctor who performs these interventions to suspect this complication in a timely manner, not delaying the diagnosis and carrying out an urgent therapeutic approach as in this case with exploratory laparotomy, finding the perforation site and carrying out the corresponding surgical management. Adding the placement of endopleural tubes for the management of significant pneumothorax and as no other alarm data were evident that required another approach, massive subcutaneous emphysema can be managed with observation. In addition, patients undergoing colonoscopy should be informed of these potential risks to enable informed decision-making and improve postprocedure follow-up and care.
